# Supplementary material for: Angelica sinensis polysaccharide as potential protectants against recurrent spontaneous abortion: focus on autophagy regulation
Source: Front Med (Lausanne). 2025 Jan 15;12:1522503. doi: 10.3389/fmed.2025.1522503 (PMC11774876; doi:10.3389/fmed.2025.1522503)
Supplement: Supplementary material S1 — The main instruments used during the LC-MS process, along with their models/specifications and manufacturers. [file Supplementary_file_1.zip › Metabolomics sequencing data FC1.2/8.代谢通路分析/Exposure_Ctrl/all/KEGG/mmu04666.html]

KEGG PATHWAY: Fc gamma R-mediated phagocytosis - Mus musculus (house mouse)


# Fc gamma R-mediated phagocytosis - Mus musculus (house mouse)


[
Pathway menu
| Organism menu
| Pathway entry
| Show description
| Download
| Help
]

Phagocytosis plays an essential role in host-defense mechanisms through the uptake and destruction of infectious pathogens. Specialized cell types including macrophages, neutrophils, and monocytes take part in this process in higher organisms. After opsonization with antibodies (IgG), foreign extracellular materials are recognized by Fc gamma receptors. Cross-linking of Fc gamma receptors initiates a variety of signals mediated by tyrosine phosphorylation of multiple proteins, which lead through the actin cytoskeleton rearrangements and membrane remodeling to the formation of phagosomes. Nascent phagosomes undergo a process of maturation that involves fusion with lysosomes. The acquisition of lysosomal proteases and release of reactive oxygen species are crucial for digestion of engulfed materials in phagosomes.


##### Option

Scale:


100%

Image resolution:


 High

##### Search

##### ID search

##### Color


KGML

Image (png) file 1x
